# Influence of Colonies’ Morphological Cues on Cellular Uptake Capacity of Nanoparticles

**DOI:** 10.3389/fbioe.2022.922159

**Published:** 2022-05-31

**Authors:** Siyuan Huang, Qi Su, Xiaoqiang Hou, Kuankuan Han, Shufang Ma, Bingshe Xu, Yingjun Yang

**Affiliations:** ^1^ Materials Institute of Atomic and Molecular Science, Shaanxi University of Science and Technology, Xi’an, China; ^2^ School of Materials Science and Engineering, Shaanxi University of Science and Technology, Xi’an, China; ^3^ Department of Critical Care Medicine, Shanghai General Hospital, Shanghai Jiao Tong University School of Medicine, Shanghai, China

**Keywords:** morphological cues, micropattern, cellular uptake, nanoparticles, colony

## Abstract

High transmembrane delivery efficiency of nanoparticles has attracted substantial interest for biomedical applications. It has been proved that the desired physicochemical properties of nanoparticles were efficient for obtaining a high cellular uptake capacity. On the other hand, biophysical stimuli from *in situ* microenvironment were also indicated as another essential factor in the regulation of cellular uptake capacity. Unfortunately, the influence of colony morphology on cellular uptake capacity was rarely analyzed. In this study, micropatterned PDMS stencils containing circular holes of 800/1,200 μm in diameter were applied to control colonies’ size. The amino-modified nanoparticles were cocultured with micropatterned colonies to analyze the influence of colonies’ morphology on the cellular uptake capacity of nanoparticles. Consequently, more endocytosed nanoparticles in larger colonies were related with a bigger dose of nanoparticles within a larger area. Additionally, the high cell density decreased the membrane–nanoparticles’ contacting probability but enhanced clathrin-mediated endocytosis. With these contrary effects, the cells with medium cell density or located in the peripheral region of the micropatterned colonies showed a higher cellular uptake capacity of nanoparticles.

## Introduction

Recently, various nanoparticles were designed and prepared for biomedical applications (Li et al., 2019; Li et al., 2021; Yang et al., 2021). Efficient cellular uptake of nanoparticles (NPs) is recognized as the prime condition for the achievement of high-yield therapeutic efficacy (Iversen et al., 2011; Liang et al., 2020). Tuning the physicochemical properties of NPs is an approachable route to improving the cellular uptake capacity (Oh and Park, 2014; Li et al., 2021). However, the optimization of NPs’ surface properties may lead to the cytotoxicity of NPs (Lewinski et al., 2008). On the other hand, cell–NP interaction and cellular uptake processes were also efficient for the regulation of cellular uptake capacity (Adjei et al., 2014). Therefore, novel techniques depending on the modulation of cell behaviors were required to avoid side effects of NP properties’ optimization. Previous evidence demonstrated that the biophysical stimuli from *in situ* microenvironment can be applied to manipulate the cellular uptake process (Huang et al., 2017). It is worth noting that physical boundary conditions were critical in regulating cell behaviors (Yang et al., 2018; Wang et al., 2021a). For instance, the effects of a single cell’s morphological cues on cell functions were revealed by micropatterning techniques (Yang et al., 2019b; Wang et al., 2021b). Additionally, the significant influence of spatial factors on colonies’ tumorigenesis (Lee et al., 2016; Lee et al., 2020) and gastrulation (Deglincerti et al., 2016; Morgani et al., 2018) has been explored by many studies. All of these results indicated the importance of morphological cues in regulating the functions of a cell or colony. Recently, the relationship between cellular uptake capacity and single cell morphology has been illustrated (Wang et al., 2016; Yang et al., 2019a; Wang et al., 2022). Whereas, in scientific research and clinical practice, a multicellular colony was more common than single-cell status. Unfortunately, even the influence of morphological cues on colonies’ behaviors was explained, and their effects on colonies’ uptake capacity were still unclear. In this case, elucidating the relationship between colonies’ morphological cues and the cellular uptake capacity of NPs will provide some essential information for the improvement of NPs’ uptake efficacy through regulating physical boundary conditions.

In this study, the effects of colonies’ morphology on the cellular uptake capacity of NPs were investigated by using micropatterned PDMS stencils. The PDMS stencils containing circular micro-holes of 0.8 and 1.2 mm in diameter were prepared to control colonies’ size. The melanoma cells with different seeding densities were cultured on the stencils to form micropatterned colonies with different sizes and cell densities. Then, the amino-modified fluorescence polystyrene NPs were incubated with micropatterned colonies to investigate the influence of colonies’ morphology on the cellular uptake capacity. Firstly, the distribution of cells and endocytosed NPs in micropatterned colonies was observed and analyzed. To explore the reason for morphological cues affecting cellular uptake capacity, the cell morphology and cytoskeleton structure were also characterized.

## Materials and Methods

### Preparation of PDMS Stencils

The stencils were prepared by a simple punching process. In detail, the PDMS film with 100 μm in thickness was commercially purchased from Hangzhou Bald Advanced Materials Technology Co., Ltd. The PDMS films were firstly cut into a circular shape of 14 mm in diameter. Then, the holes of 0.8 mm or 1.2 mm in diameter were manufactured on the circular PDMS film by using a specific puncher. Before cell seeding, the prepared stencils were firstly sterilized by immersing in 70% ethanol for 20 min and rinsing in PBS solution twice. Finally, the stencils were placed in a 24-well plate for cell culture.

### Cell Culture

Melanoma cells (B16) were purchased from Procell Lifer Science & Technology Co., Ltd. and subcultured in DMEM medium (Mishu (Xi’an) Biotechnology Co., Ltd.) supplied with 10% FBS (Biological Industries Israel Beit Haemek Ltd.) and 1% penicillin–streptomycin (Mishu (Xi’an) Biotechnology Co., Ltd.). A 1 ml cell suspension with 4 × 10^4^, 6 × 10^4^, and 8 × 10^4^ cells/ml in cell density was seeded in each well. After being cultured in a humidified CO_2_ incubator for 6 h, the medium with suspending cells was refreshed. Then, the samples were further incubated for 18 h for the following experiments.

### Nuclei Staining and Cell Density Analysis

After cell seeding for 24 h, the samples were rinsed with prewarmed PBS and fixed with 4% cold paraformaldehyde (Shanghai Aladdin Biochemical Technology Co., Ltd.) for 10 min. Then, the samples were permeabilized by 1% Triton X-100 (Shanghai Aladdin Biochemical Technology Co., Ltd.) for 2 min and stained by 1‰ DAPI (Shandong Sparkjade Scientific Instruments Co., Ltd.) in PBS for 10 min. The fluorescence of DAPI was observed and recorded by a fluorescence microscope (MF52-N, Guangzhou Micro-shot Technology Co., Ltd.). The fluorescence images were applied to analyze cell distribution. To count cells in different regions, the colony was firstly separated into the peripheral region and central region. To get the same area of the peripheral region and central region, the radius of the central region was set as 0.28 mm for micropatterned colonies with 0.8 mm in diameter and 0.42 mm for colonies with 1.2 mm in diameter. The cell numbers at the peripheral or central regions were counted from DAPI staining fluorescence images by analyzing the particles processed in ImageJ. More than 50 fluorescent images were applied to get heat maps, and five representative fluorescence images were analyzed to get quantitative data.

### Cellular Uptake Capacity Analysis

After the cell seeding process for 24 h, the medium was replaced by a fresh medium with 1% amino-modified fluorescent PS NPs (100 nm, Xi’an ruixi Biological Technology Co., Ltd.) and further incubated for another 24 h in a humidified CO_2_ incubator. Then, the samples were harvested and incubated with 0.4% trypan blue (Shanghai Aladdin Biochemical Technology Co., Ltd.) for 5 min to quench the fluorescence of extracellular NPs. Finally, the samples were fixed with 4% paraformaldehyde and stained with 1‰ DAPI. The fluorescence of NPs and DAPI was observed and recorded by fluorescence microscopy. The percentage and fluorescence intensity of NP-positive cells were calculated to evaluate the cellular uptake capacity. To define NP-positive cells, the integrated gray value (IGV) and area (A) of each cell were calculated by ImageJ. To get the corrected IGV, the region without cell attachment was selected as the background to calculate IGV_background_ and A_background_. Then, the corrected IGV was calculated by (IGV/A-IGV_background_/A_background_)×A. The NP-positive cells were defined as the cells with corrected IGV two times higher than the IGV_background_. The percentage of NP-positive cells was calculated by the number of NP-positive cells divided by the total cell number. The corrected IGV of nanoparticle-positive cells was also recorded to evaluate the cellular uptake capacity. More than 30 fluorescent images of each group were analyzed.

### Actin and Nuclei Staining

After cells were cultured within stencils for 24 h, the samples were fixed by 4% paraformaldehyde for 10 min and permeabilized by 1% Triton X-100 for 2 min at room temperature. Then, the samples were blocked with 2% bovine serum albumin (BSA, Shanghai Aladdin Biochemical Technology Co., Ltd.) in PBS for 30 min at room temperature. Then, actin was stained by incubating the samples with Alexa Fluor-594 phalloidin (Beijing Solarbio Science & Technology Co., Ltd.) at a dilution ratio of 1:40 in PBS for 20 min at room temperature. Nuclei were stained with 1‰ DAPI at room temperature in the dark for 10 min. After being washed with PBS three times, the fluorescence images of each sample were observed and recorded by a fluorescence microscope. Fifty fluorescent images were applied to obtain heat maps.

### Blebbistatin and Dynasore Treatment

Blebbistatin (Shanghai Aladdin Biochemical Technology Co., Ltd.) and dynasore (Shanghai Aladdin Biochemical Technology Co., Ltd.) were applied to disturb the cytoskeleton organization and inhibit the dynamin activity. In detail, after the cell was cultured on stencils for 16 h, the medium was refreshed by a cell-cultured medium with 1 ng/ml blebbistatin or 40 μM dynasore. After further culturing for 8 h, the medium was replaced by a fresh medium with 1% amino-modified fluorescent NPs and 1 ng/ml blebbistatin or 40 μM dynasore. After further culturing in a humidified CO_2_ incubator for another 24 h, the samples were harvested and treated with 0.4% trypan blue for 5 min. Before fluorescence observation, the samples were stained with DAPI. The percentage and fluorescence intensity of NP-positive cells were also analyzed as previously described. More than 30 fluorescent images were analyzed.

### Statistical Analysis

The significant difference among samples was performed using a one-way analysis of variance (ANOVA) with Tukey’s *post hoc* test for multiple comparisons. The data are presented as means ± standard deviations (SDs). It is considered to be a statistically significant difference when *p* < 0.05.

## Results

### Preparation and Characterization of Stencils

**FIGURE 1:**
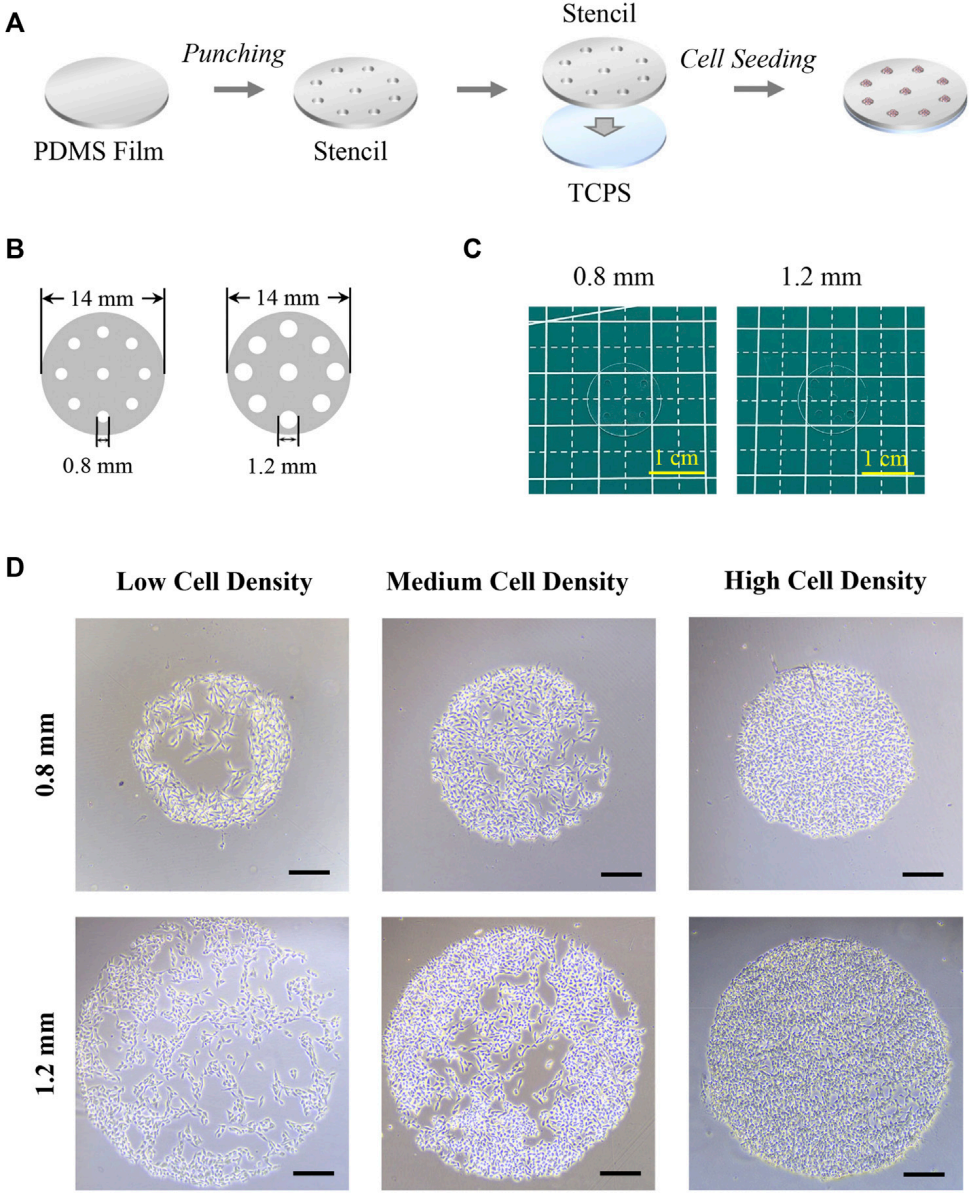
Preparation of PDMS stencils and morphology of micropatterned colonies. **(A)** Scheme of PDMS stencil’s preparation and application in cell culture. **(B)** Illustration of PDMS stencils’ structure. **(C)** Photography of PDMS stencils. **(D)** Microscopic images of micropatterned melanoma colonies. Scale bar: 200 μm.

As shown in Figure 1A, PDMS stencils were prepared by the punching process and applied for cell culture. The micro-holes with 0.8 mm or 1.2 mm in diameter were fabricated on a circular PDMS film with 14 mm in diameter by using a specific puncher (Figures 1B, C). The diameter of the holes was 849.5 ± 33.9 μm and 1,273.7± 62.6 μm. After sterilization, the PDMS stencils tightly adhered to TCPS (tissue culture polystyrene) surfaces in 24-well plates for the following cell experiments. Since the PDMS film’s hydrophobic properties do not encourage cell adhesion (Subramaniam and Sethuraman, 2014), cells tended to adhere to the TCPS surface that bore micro-holes from the PDMS stencils (Choi et al., 2012). After the cell was cultured with PDMS stencils, colonies were formed within micro-holes of the stencil and the morphology of the colonies was controlled by PDMS stencils (Figure 1D). After cell adhesion, the unadhered cells were easily removed by the medium refreshing process. Additionally, the stencil-associated micropatterning method had no significant influence on cell viability (Supplementary Figure S1).

### Cell Distribution

**FIGURE 2:**
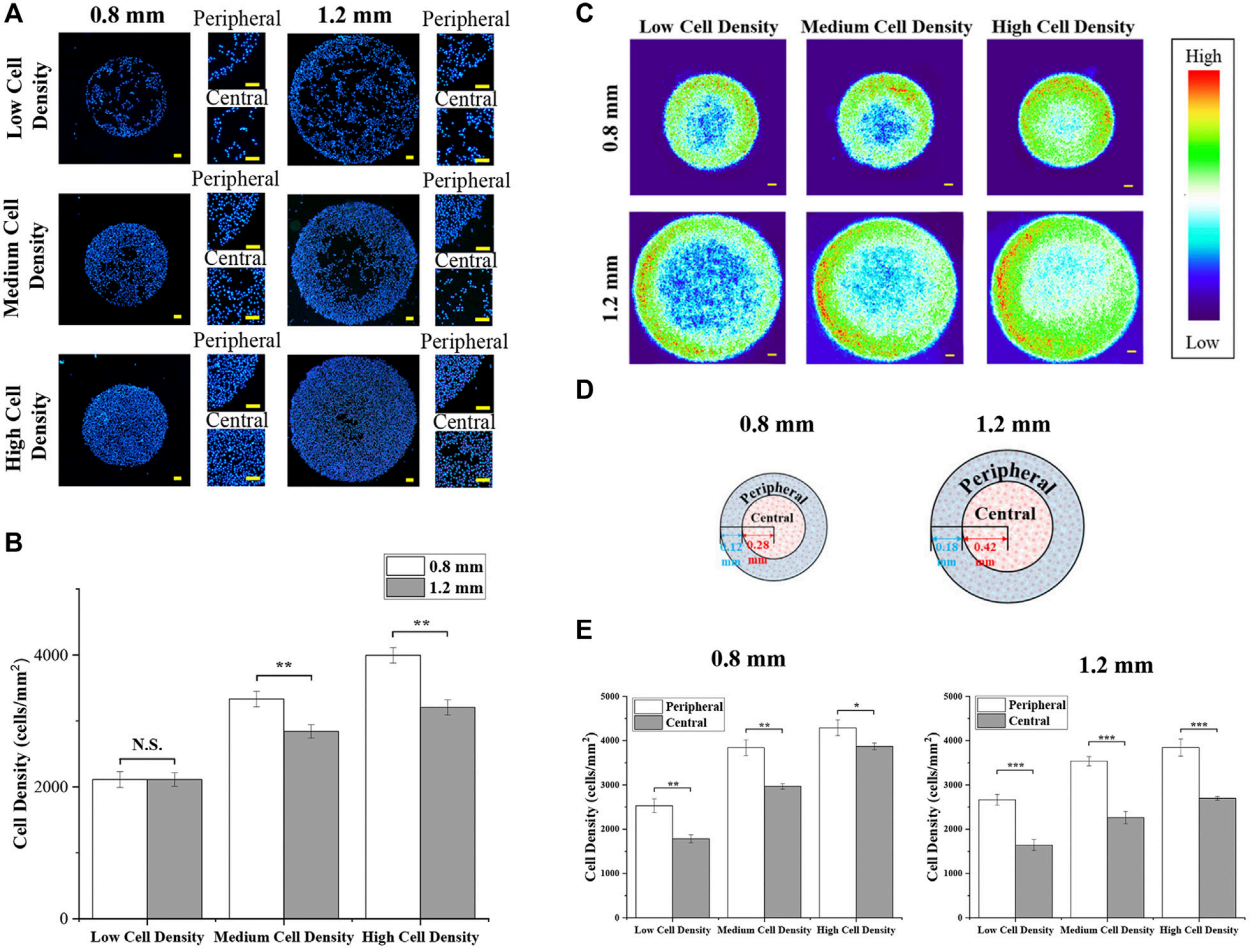
Cell distribution of micropatterned colonies. **(A)** Distribution of DAPI-stained nuclei in colonies with different diameters and cell densities. Scale bar: 50 μm. **(B)** Cell density of micropatterned colonies. **(C)** Heat maps of DAPI-stained nuclei. Scale bar: 50 μm. **(D)** Illustration of the peripheral region and central region in micropatterned colonies. **(E)** Cell density of the defined peripheral and central regions in micropatterned colonies with different diameters and cell densities. Data are presented as means ± SDs (*n* = 5). *p < 0.05, **p < 0.01, ***p < 0.001.

After the formation of micropatterned colonies, the diameter of the micropatterned melanoma colonies was 835.9 ± 14.1 μm and 1,262.2 ± 18.4 μm. Then, the nuclei were stained to characterize the cell distribution. As shown in Figure 2A, the density of melanoma cells was controlled by seeding density. With few cells seeding, there was no significant difference in cell density between different sizes of colonies (Figure 2B). With increased seeding density, the cell density of colonies with 0.8 mm in diameter was higher than the colonies with 1.2 mm in diameter (Figure 2B).

Furthermore, cells predominantly adhered at the peripheral region of micropatterned colonies. To observe more clearly, more than 50 fluorescent images were applied to create heat maps (Figure 2B). The heat maps showed the same phenomenon that melanoma cells concentrated in the peripheral region of micropatterned colonies, especially at low cell density. To quantitatively analyze the cell density at a different region of colonies, the central and peripheral regions with the same area were set, as shown in Figure 2C. As Figure 2Dshows thequantitative results, the cell density in the peripheral region was significantly higher than in the central region. In addition, with cell density increased, cells were more homogeneously distributed within smaller colonies.

### Cellular Uptake Capacity

**FIGURE 3:**
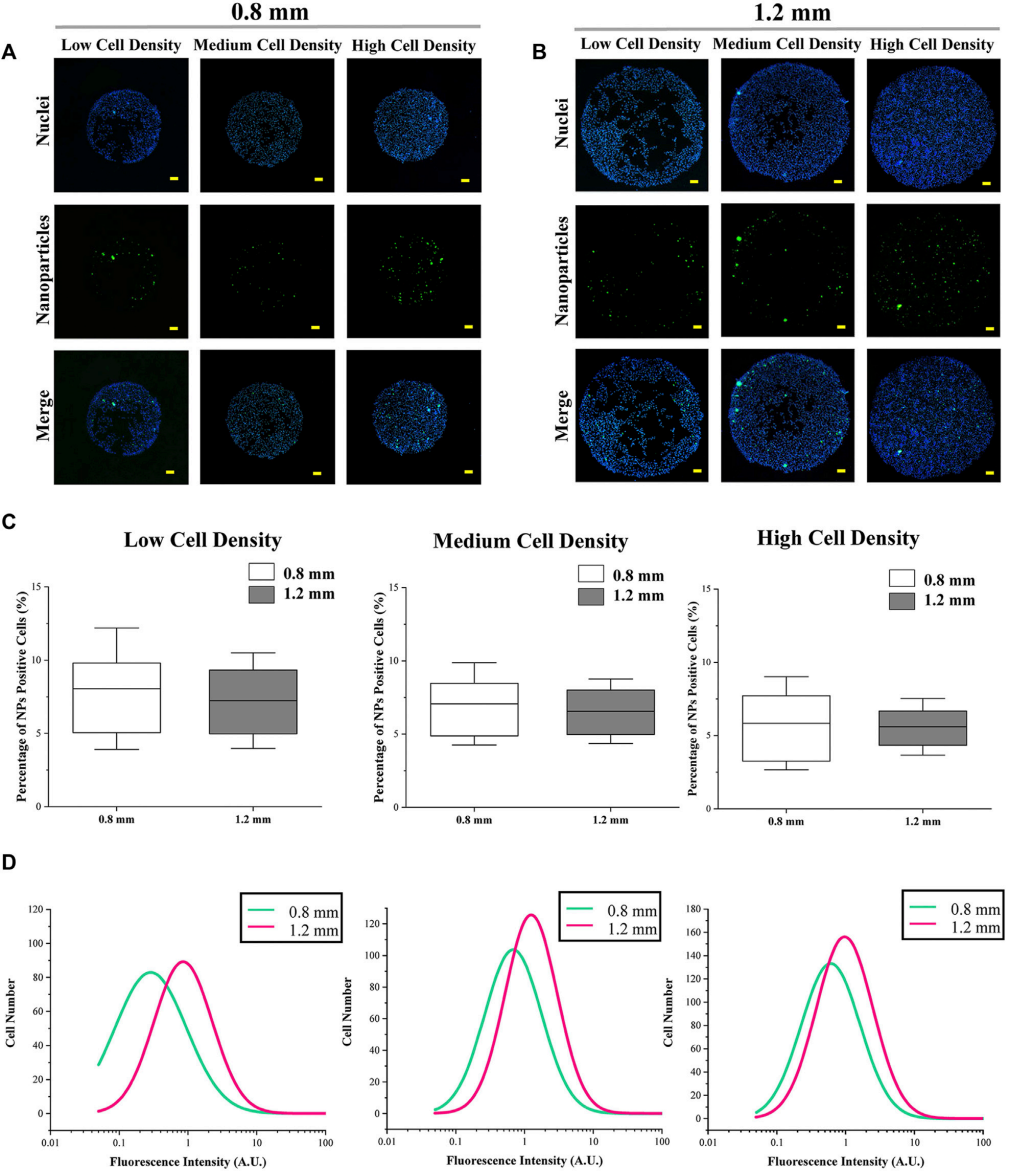
Cellular uptake capacity of micropatterned colonies with different sizes. **(A,B)** Representative images of fluorescent NPs and nuclei. Scale bar: 50 μm. **(C)** Percentage of NP-positive cells. Data are presented as means ± SDs (*n* > 30). **(D)** Fluorescence intensity of NP-positive cells (*n* > 3,000).

The cellular uptake of amino group-modified fluorescent PS NPs is shown in Figures 3A,B. The cellular uptake capacity was indicated by the percentage and fluorescence intensity of NP-positive cells. The percentage of NP-positive cells was related to the ratio of cells, which could uptake NPs, and the fluorescence intensity of NP-positive cells was applied to indicate the amount of endocytosed NPs in each cell. In the results, colony size did not have a significant effect on the percentage of NP-positive cells (Figure 3C). On the other hand, the fluorescence intensity of cells in micropatterned colonies with 1.2 mm in diameter was higher than in the smaller colonies (*d* = 0.8 mm) (Figure 3D).

**FIGURE 4:**
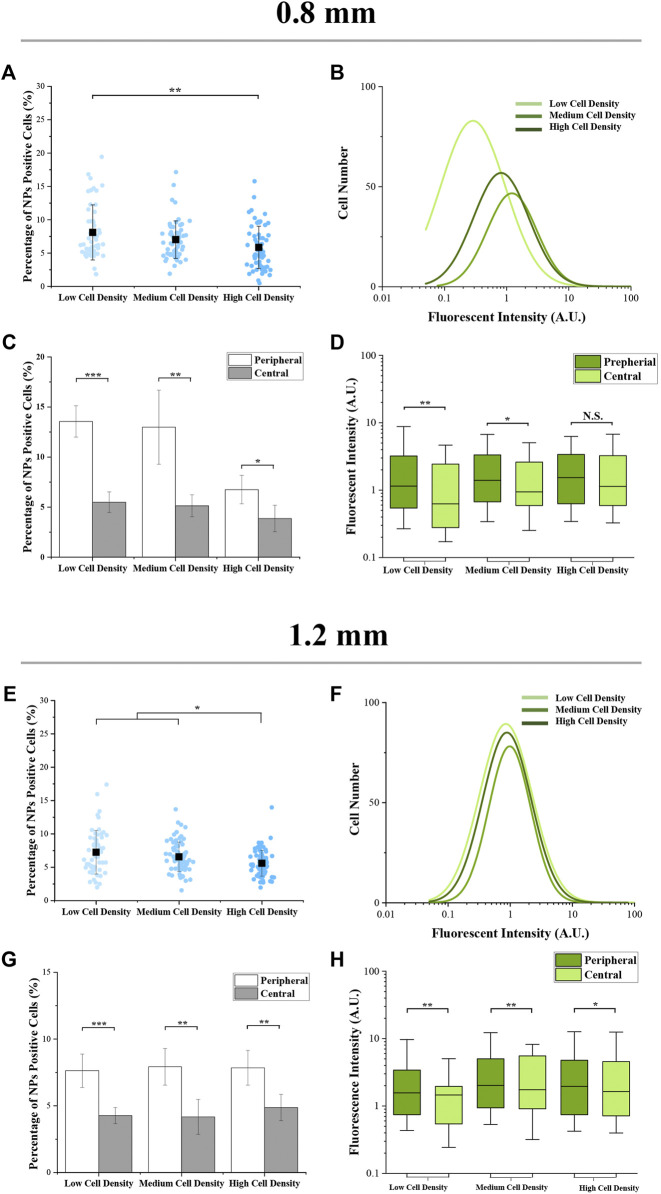
Cellular uptake capacity of micropatterned colonies with different regions. **(A,E)** Percentage of NP-positive cells within whole colonies. Data are presented as means ± SDs (n > 30). **(B,F)** Distribution curves of NP-positive cells’ intensity (n > 3,000). **(C,G)** Percentage of NP-positive cells at the peripheral and central regions. Data are presented as means ± SDs (*n* = 5). **(D,H)** Fluorescence intensity of NP-positive cells at the peripheral and central regions. Data are presented as means ± SDs (n > 100). *p < 0.05, **p < 0.01, ***p < 0.001.

Furthermore, within the colonies having the same size, as the cell seeding density increased, the percentage of NP-positive cells was decreased (Figures 4A,E. It was interesting that the fluorescence intensity of cells with a medium density was higher than the cells with low or high density (Figures 4B,F ). On the other hand, for the results of spatial factor that regulated the cellular uptake capacity, the percentage and fluorescence intensity of NP-positive cells (Figures 4C,G and Figures 4D,H) in the peripheral region were significantly higher than the cells located in the central region. To explore the reason for these phenomena, the cytoskeleton of melanoma cells was analyzed.

### Structure of Cytoskeleton

**FIGURE 5:**
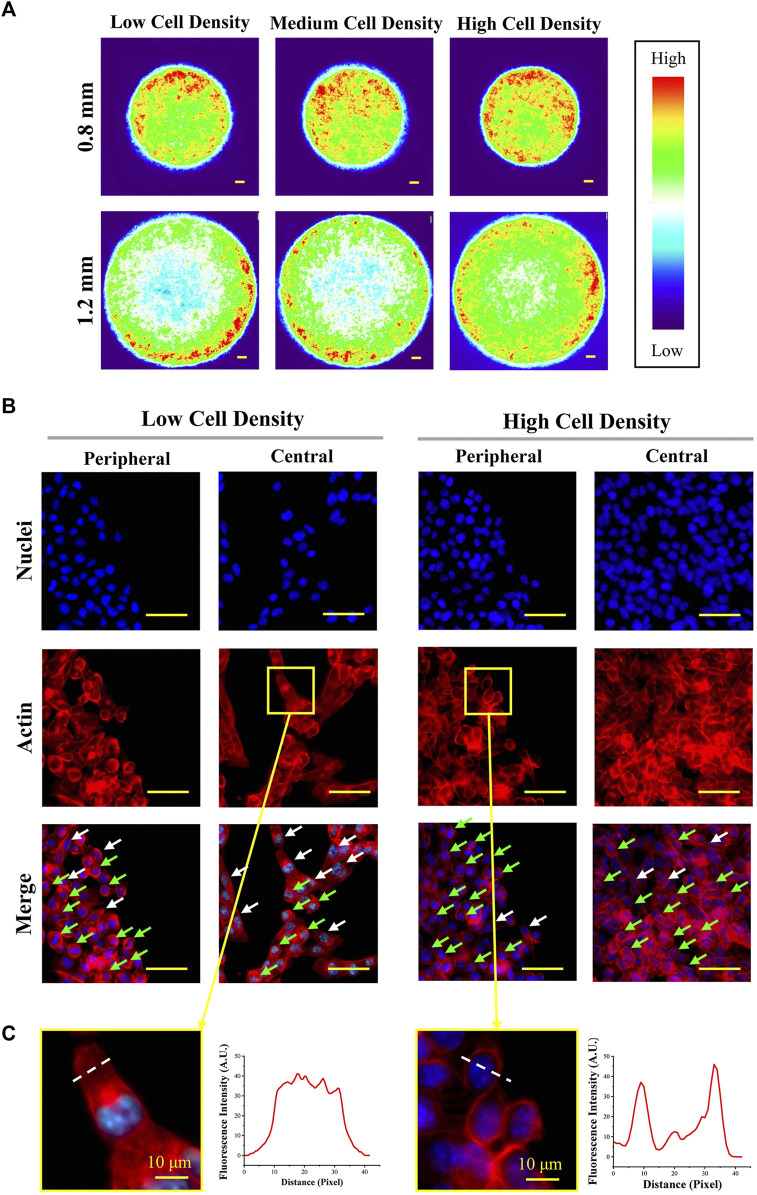
Structure of actin at the peripheral and central regions of colonies. **(A)** Heat maps of actin-stained fluorescent images. Scale bar: 50 μm. **(B)** Representative magnified fluorescent images of stained nuclei and actin (*d* = 1.2 mm). Scale bar: 50 μm. **(C)** Representative images and fluorescence intensity curves of spindle-shaped and circular-shaped melanoma cells (*d* = 1.2 mm). Scale bar: 10 μm.

Since previous studies have demonstrated that the cellular uptake capacity is tightly related to the structure of the cytoskeleton (Wang et al., 2021a; Wang et al., 2021b; Wang et al., 2021c), the actin structure was characterized in this study. The structure of the cytoskeleton was analyzed by actin-stained fluorescence images (Supplementary Figure S2). The heat maps of actin (Figure 5A) revealed that actin was concentrated in the peripheral region of micropatterned colonies. This result had a good agreement with cell uneven distribution. As shown in the magnified fluorescent images (Figure 5B), more spindle-shaped cells (white arrow) were observed in the central region of the micropatterned colony with low cell seeding density. In contrast, more circular-shaped cells (green arrow) were found in peripheral regions with high cell density. In addition, actin was concentrated in the cortical region in circular-shaped cells and homogeneously distributed in spindle-shaped cells (Figure 5C).

### Influence of Cytoskeleton on Cellular Uptake Capacity

**FIGURE 6:**
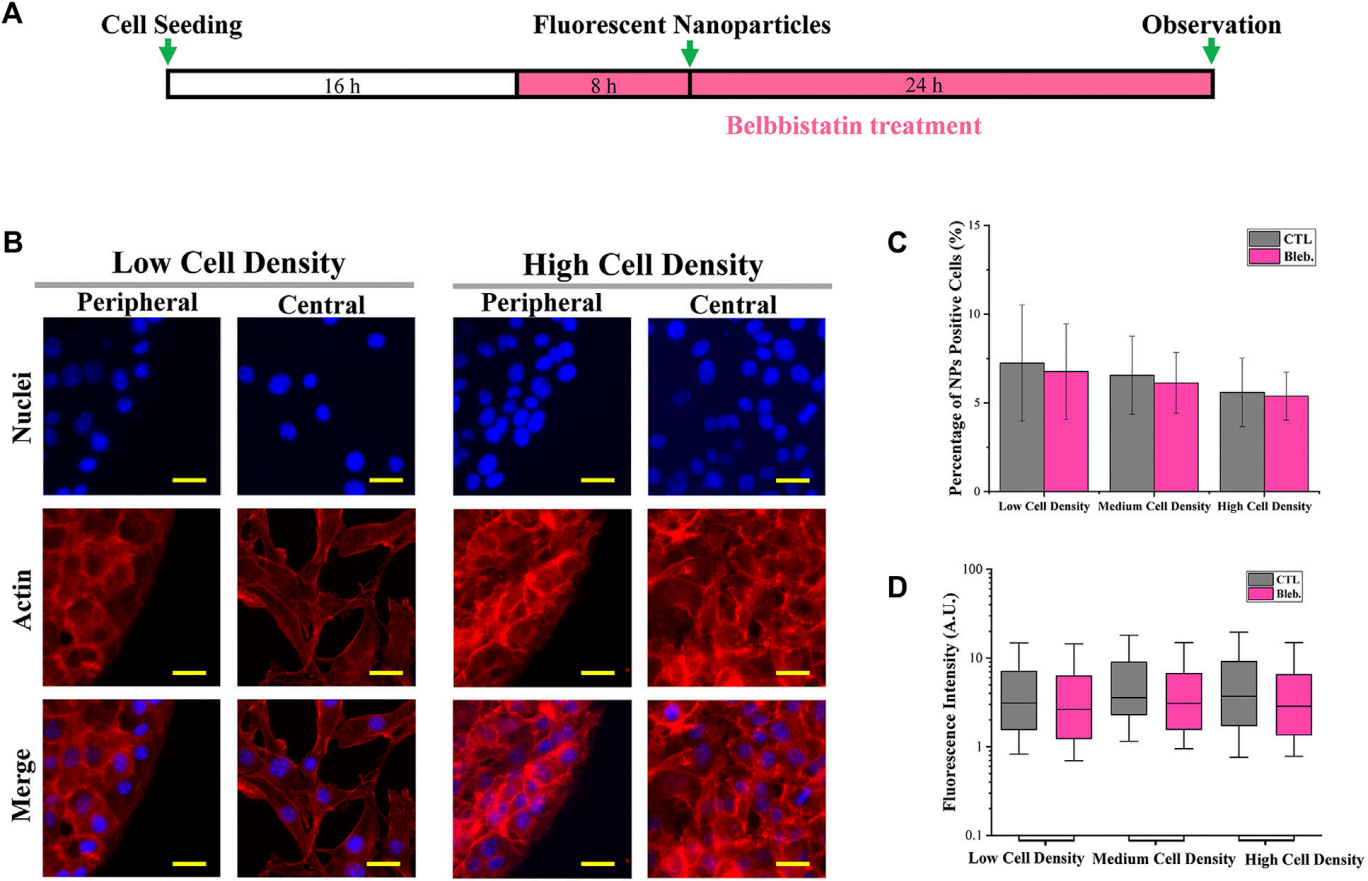
Influence of the cytoskeleton structure on cellular uptake capacity. **(A)** Scheme of blebbistatin treatment. **(B)** Structure of actin after blebbistatin treatment (*d* = 1.2 mm). Scale bar: 50 μm. **(C)** Percentage of NP-positive cells after blebbistatin treatment (*d* = 1.2 mm). Data are presented as means ± SDs (*n* > 30). **(D)** Fluorescence intensity of NP-positive cells after blebbistatin treatment (1.2 mm). Data are presented as means ± SDs (*n* > 300).

To explore the function of cytoskeleton in the cellular uptake process, the cytoskeleton was disrupted. As shown in Figure 6A, actin was disturbed by blebbistatin before and during cellular uptake experiments. After blebbistatin treatment, there was no significant influence on the actin organization of spindle-shaped cells. In contrast, actin in the cortical region of cells with high density disappeared and was randomly distributed within the cytoplasm. In addition, the percentage (Figure 6C; Supplementary Figure S3A) and fluorescence intensity (Figure 6D; Supplementary Figure S3B) of NP-positive cells were slightly decreased after blebbistatin treatment.

### Influence of Dynamin on Cellular Uptake Capacity

**FIGURE 7:**
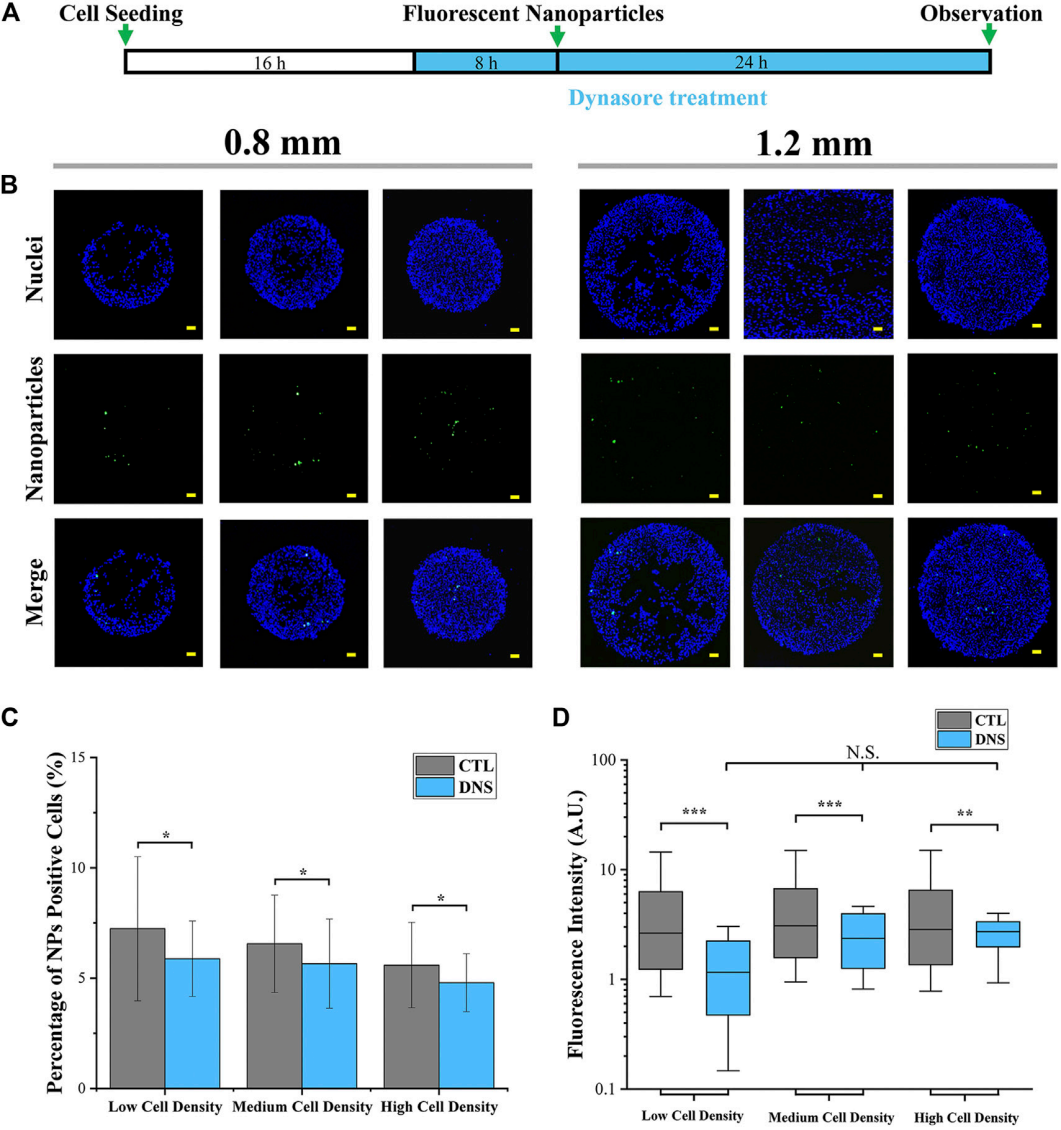
Influence of dynamin on cellular uptake capacity. **(A)** Scheme of dynasore treatment. **(B)** Representative fluorescent images of cellular uptake after dynasore treatment. Scale bar: 50 μm. **(C)** Percentage of NP-positive cells after dynasore treatment (1.2 mm). Data are presented as means ± SDs (*n* > 30). **(D)** Fluorescence intensity of NP-positive cells after dynasore treatment (1.2 mm). Data are presented as means ± SDs (*n* > 300). *p < 0.05, ***p < 0.001.

In addition, at the final step of clathrin-mediated endocytosis (CME), cortical actin is collaborated with dynamin to separate clathrin-coated pits from the plasma membrane (Merrifield et al., 2002; Grassart et al., 2014). It means that cortical actin is functionalized not only at the process of endocytic membrane’ invagination but also at the endocytic vesicles’ separation. Therefore, the function of cortical actin and dynamin in the regulation of cellular uptake capacity was also investigated by the inhibition of dynamin activity. In this case, dynasore was applied to inhibit dynamin activity (Macia et al., 2006). As shown in Figure 7A, dynasore was incubated with colonies before and during cellular uptake experiments. Quantitative data revealed that the percentage (Figure 7C; Supplementary Figure S4A) and fluorescence intensity (Figure 7D; Supplementary Figure S4B) of NP-positive cells were significantly decreased after dynasore treatment. In addition, the fluorescence intensity has no significant difference with different cell seeding densities after dynasore treatment (Figure 7D).

## Discussion

In this study, micropatterned PDMS stencils were applied to control the morphology of melanoma colonies. After micropatterned colonies formed, the size and morphology of colonies were easily controlled by PDMS stencils and the cell density was regulated by the cell seeding process. For each colony, the cells were predominately located in the peripheral region. This phenomenon was similar to the cells cultured in microwell plates. This uneven distribution of cells has been explained by the effect of the meniscus (Freshney, 2010).

Then, the results of cellular uptake capacity and morphological cues of colonies were comprehensively analyzed. Firstly, the fluorescence intensity of NP-positive cells in a larger colony was higher than the cells in a smaller colony (Figure 3D). In common sense, with homogeneously distributed NPs, the colonies with a larger spreading area have a higher contact probability with NPs. Thus, this phenomenon was considered to be the result of a bigger dose of NPs. In addition, the role of cell density and spatial factors in the regulation of cellular uptake capacity was also analyzed. With cell density increased, more concentrated cells showed circular morphology and a smaller spreading area (Figure 5B). This smaller spreading area induced a lower contact probability between the plasma membrane and NPs and finally decreased the percentage of NP-positive cells (Figures 4A,E) (Wang et al., 2016; Khetan et al., 2019). In contrast, the fluorescence intensity of positive cells with medium density was higher than the cells with low density (Figures 4B,F). It means that even if the percentage of NP-positive cells decreased, the cellular uptake capacity of each individual cell was enhanced by higher cell density. These controversial results indicated that the increased cell density could enhance the cellular uptake capacity of an individual cell but decrease the contacting probability between the cytoplasm membrane and NPs. Moreover, this inference was also supported by the decreased fluorescence intensity of NP-positive cells with the highest cell density.

Furthermore, with the result of uneven cell distribution in micropatterned colonies (Figure 2), the concentrated cells in the peripheral region have the highest percentage and fluorescence intensity of NP-positive cells (Figure 4). It also indicated that the higher cellular uptake capacity was related to higher cell density. In this case, the structure and functions of the cytoskeleton were characterized. The cells with higher density showed more circular-shaped cells with the typical cortical actin. As previously reported, the cortical actin was critical for invagination of the endocytic membrane in CME (Galletta et al., 2010; Chaudhuri et al., 2011). Additionally, some research already revealed that CME is a primary approach for the cellular uptake of amino-modified NPs (Jiang et al., 2010). Thus, cortical actin was considered a critical factor in the regulation of the cellular capacity of NPs. Therefore, the higher cell density-enhanced cellular uptake capacity benefited from cortical actin-accelerated CME in circular-shaped cells with high cell density. To verify the functions of cortical actin in the regulation of cellular uptake capacity, the actin organization was disrupted by blebbistatin (a specific myosin II inhibitor that can reduce cortical actin-mediated cortex tension) (Lu et al., 2008; Tinevez et al., 2009). For the results, with the disappearance of cortical actin, cellular uptake capacity was decreased (Figure 6). This could be further evidence to support the fact that the higher cellular uptake capacity of concentrated cells benefited from the cortical actin-enhanced CME. Additionally, since the cortical actin also takes effect on dynamin during the endocytic membrane’s separation (Loebrich, 2014), the dynamin was suppressed by dynasore treatment. After dynasore treatment, the significantly decreased cellular uptake capacity was independent of cell density (Figure 7D). The effects of dynamin demonstrated the improved cellular uptake in higher cell density was mainly contributed by cortical actin assisted membrane separation.

In summary, with a bigger dose of NPs, larger colonies endocytosed more NPs. In addition, the contrary effects of high cell density on the cellular uptake of NPs were revealed. Firstly, the concentrated cells with a circular shape showed typical cortical actin that can accelerate CME. On the other hand, the negative effect of high cell density on the cellular uptake capacity was associated with a decreased contacting probability between NPs and cytoplasm membrane. For these reasons, cells with higher density (intermediate seeding density or located at the peripheral region of micropatterned colonies) showed a higher cellular uptake capacity of NPs.

## Conclusion

In this research, PDMS stencils were prepared and applied to control the morphologies of melanoma colonies. Subsequently, the influence of morphological cues on cellular uptake capacity has been revealed. The results indicated that more endocytosed NPs in larger colonies were related to a bigger dose of NPs within larger areas. In addition, with cell density increased, the cellular uptake capacity was simultaneously enhanced by the cortical actin-accelerated CME and was inhibited by the decreased contacting probability of NPs and cytoplasm membrane. As a result, cells with intermediate seeding density or located in the peripheral region of micropatterned colonies showed the highest cellular uptake capacity of NPs.

## Data Availability

The raw data supporting the conclusions of this article will be made available by the authors without undue reservation.
