# Supplementary material for: Influence of Colonies’ Morphological Cues on Cellular Uptake Capacity of Nanoparticles
Source: Front Bioeng Biotechnol. 2022 May 31;10:922159. doi: 10.3389/fbioe.2022.922159 (PMC9194857; doi:10.3389/fbioe.2022.922159)
Supplement: Supplementary file 1 [file DataSheet1.DOCX]

**Supplementary material:**


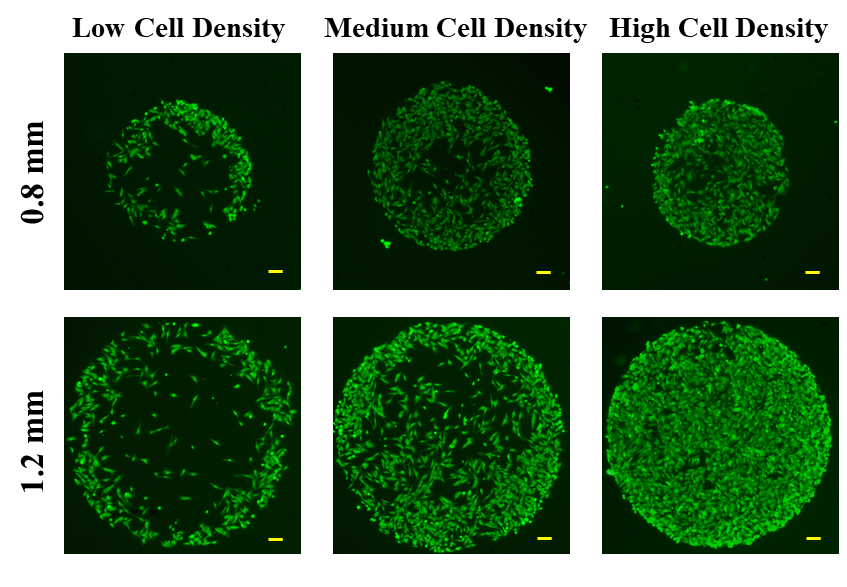


**Figure S1.** Live/Dead staining images of micropatterned colonies. Scale bar: 50 μm.


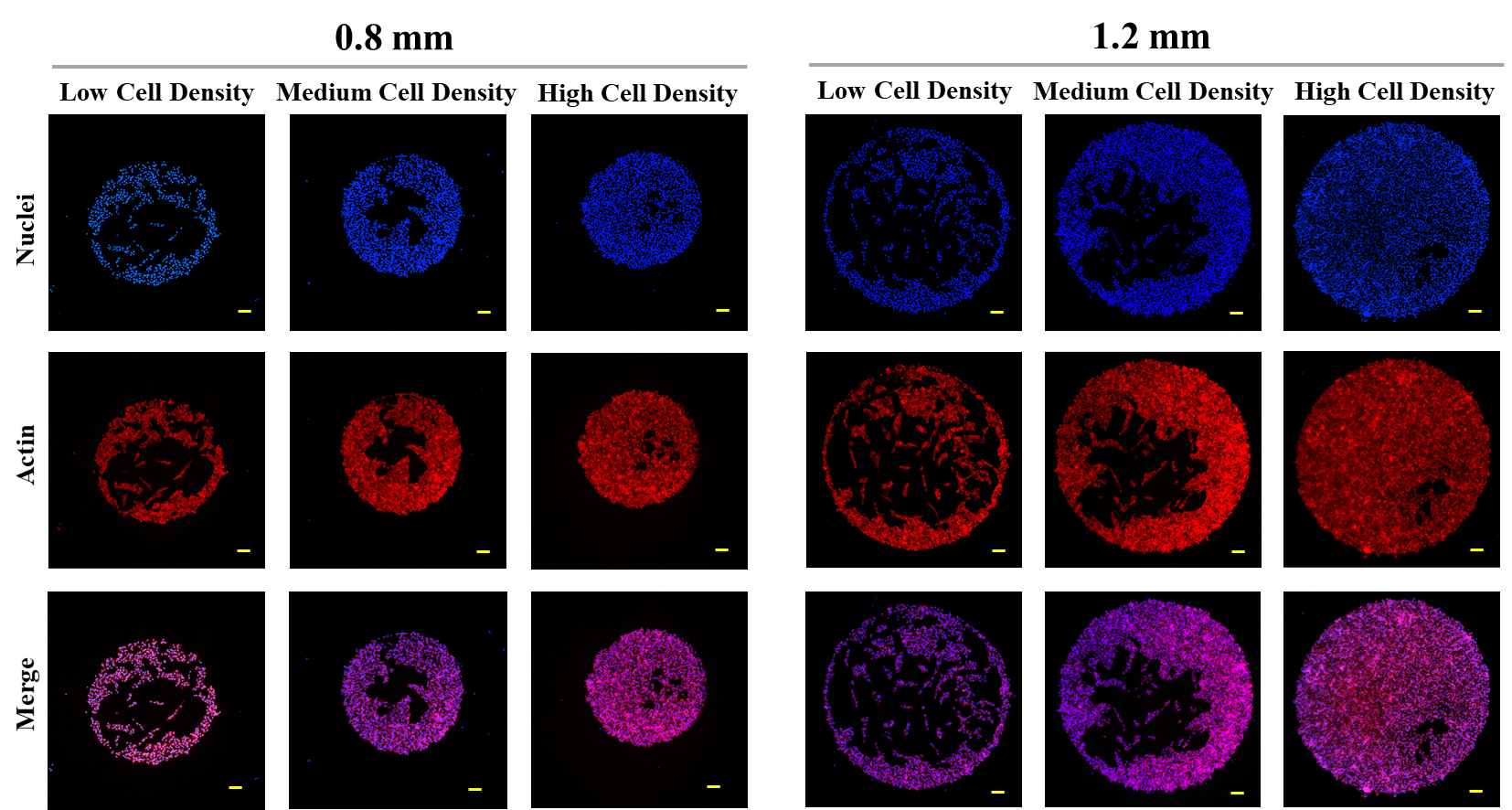


**Figure S2.** Actin/nuclei staining images of micropatterned colonies. Scale bar: 50 μm.


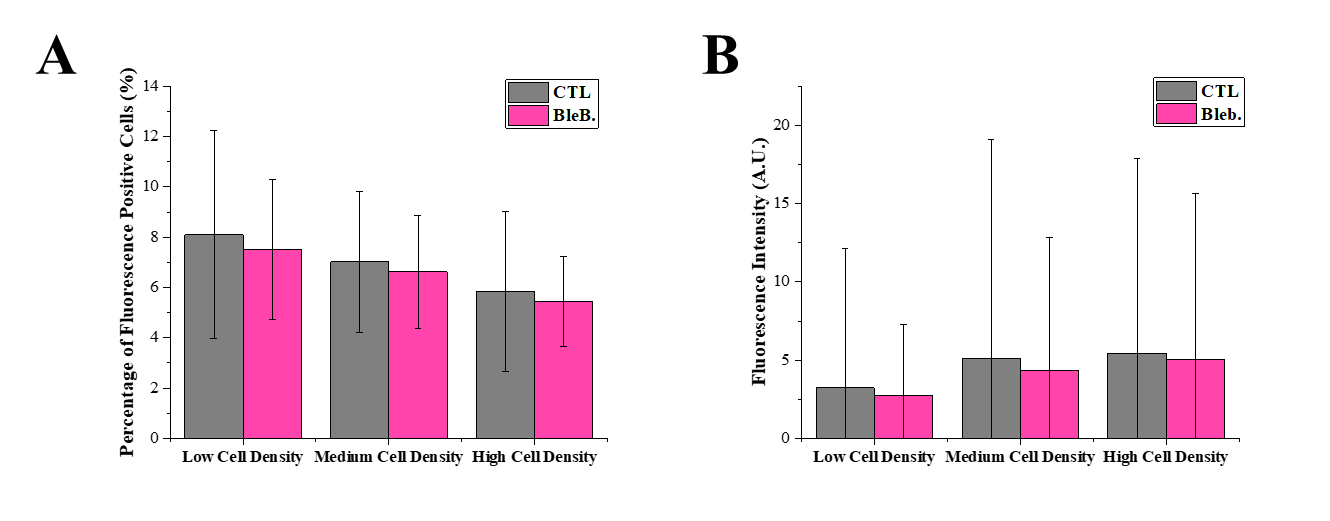


**Figure S3.** Cellular uptake capacity of micropatterned colonies after blebbistatin treatment. (A) Percentage of fluorescence positive cells after blebbistatin treatment (0.8 mm). Data are presented as means ± SDs (n>30). (B) Fluorescence intensity of positive cells after blebbistatin treatment (0.8 mm). Data are presented as means ± SDs (n>300).


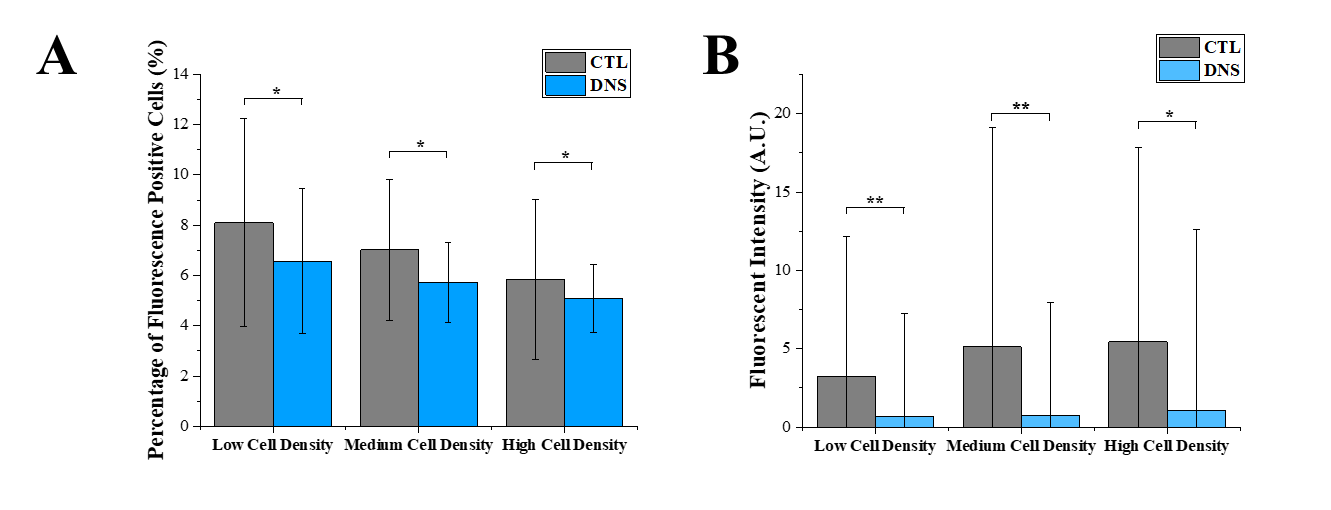


**Figure S4.** Cellular uptake capacity of micropatterned colonies after dynasore treatment. (A) Percentage of fluorescence positive cells after blebbistatin treatment (0.8 mm). Data are presented as means ± SDs (n>30). (B) Fluorescence intensity of positive cells after blebbistatin treatment (0.8 mm). Data are presented as means ± SDs (n>300). *p < 0.05, **p < 0.01.
